# Prevalence of mental health symptoms and potential risk factors among Austrian psychotherapists

**DOI:** 10.1038/s41598-024-54372-7

**Published:** 2024-02-16

**Authors:** Yvonne Schaffler, Thomas Probst, Christoph Pieh, Barbara Haid, Elke Humer

**Affiliations:** 1 Department of Psychosomatic Medicine and Psychotherapy, University for Continuing Education Krems, Krems, Austria; 2 Division of Psychotherapy, Department of Psychology, Paris Lodron University Salzburg, Salzburg, Austria; 3 Austrian Federal Association for Psychotherapy, Vienna, Austria; 4 Faculty of Psychotherapy Science, Sigmund Freud University Vienna, Vienna, Austria

**Keywords:** Psychotherapist, Mental health, Depression, Anxiety, Insomnia, Stress, Income, Physical activity, Psychology, Health care, Occupational health, Signs and symptoms

## Abstract

This research focused on the intricacies of mental health within the psychotherapist community, a domain currently understudied. A recent study suggests a better overall mental health profile for Austrian psychotherapists compared to the general population. However, a substantial proportion of psychotherapists still exhibited scores beyond the cut-off for clinically significant mental disorders, potentially undermining the efficacy of patient outcomes. A cross-sectional study was conducted between April to June 2023, enrolling a cohort of 502 Austrian psychotherapists (79.7% female; mean age: 53.4 ± 9.26 years). The analysis leveraged indicators for symptoms of depression (PHQ-2), anxiety (GAD-2), insomnia (ISI-2), stress (PSS-4), and overall well-being (WHO-5). Key correlations were revealed using multivariable logistic regressions incorporating sociodemographic, work-related, and health behavior variables. Mental health symptoms in psychotherapists were associated with lower income, lesser physical activity, employment in outpatient facilities, less experience in the field, and a smaller patient load. Notably, physical activity emerged as a significant protective component, underscoring regular exercise as a vital self-care routine for psychotherapists. Adequate income also presented as a crucial contributor to mental health stability. These findings augment our understanding of the complex dynamics influencing psychotherapists' mental health, potentially enhancing their well-being and optimizing professional output.

The nature of psychotherapeutic work inherently involves high emotional engagement, which may predispose psychotherapists to an elevated risk of developing mental disorders^1,2^. Historically, research has skewed towards the mental health of patients receiving psychotherapy, with relatively little focus on the mental well-being of the practitioners themselves. Much of the existing research has primarily concentrated on burnout^3–6^, leaving other manifestations of mental distress, such as symptoms of depression, anxiety, and insomnia, comparatively less explored^7^. This research gap is noteworthy, given the dual significance of psychotherapists' mental health—it is integral to their personal quality of life and their capacity to deliver high-quality therapeutic services^8^. The literature underscores the vital link between psychotherapists' mental health and the quality of therapeutic care, emphasizing that practitioners must be well themselves to effectively benefit patients^9^. Research indicates that patients prefer psychotherapists perceived as psychologically healthy, wise, and satisfied with life^10,11^. This not only impacts therapists' well-being but also plays a vital role in shaping positive patient perceptions and enhancing therapeutic effectiveness^12^. Recognizing practitioners as vulnerable tools, ethical obligations and codes emphasize the importance of practitioner self-care to prevent negative consequences for both practitioners and their patients^8^. Therefore, it is of considerable importance to conduct in-depth research that can shed light on the mental health status of psychotherapists and strive to understand the factors linked with their mental health vulnerabilities. These studies could play a crucial role in preventing professional impairment within psychotherapeutic services, thereby safeguarding the efficacy of patient care.

The few previous studies on mental health in mental health care professionals have been controversial^8,13,14^, with results ranging from lower^14^ to higher prevalences of mental health issues^13^ in mental health care professionals compared to the general public. A recent study from Austria carried out in spring 2022 suggests that mental healthcare professionals may have better mental health than the general population^7,15^. However, a sub-group of psychotherapists experienced poor well-being or even exceeded critical cut-offs for clinically relevant symptoms of depression, anxiety, insomnia, or moderate to high stress levels^7^. Associations with gender and age were observed, and a previous study conducted in the spring of 2020 (during the first nationwide COVID-19 lockdown in Austria) observed higher perceived stress levels in psychotherapists whose income derived solely from psychotherapy^16^. However, previous experience with remote psychotherapy, the treatment format used during the lockdown, and the changes in the patient numbers compared to the time before the lockdown were not associated with the perceived stress level^16^. Several recent studies on the Austrian general population revealed mental health associations with gender, age, income, physical activity, and smartphone usage^17–19^.

The latest study on the Austrian general population was conducted in spring 2022 and revealed that, when several sociodemographic variables were considered simultaneously, mental health symptoms were mainly associated with gender (female: increased odds for depression, male: increased odds for alcohol abuse), younger age (< 35 years) and low income (< €2000 net household income per month)^20^. Previous studies on healthcare workers^21^ and psychotherapists^3^ observed differences in the prevalence of psychological distress concerning age, region, income, gender, work settings, workload, and work experience. In general, healthcare professionals originating from nations with higher rates of human development, greater number of physicians, and increased health expenditure indicated a higher prevalence of depression, anxiety and stress^21^. Burnout symptoms seem to be more prevalent in younger psychotherapists and those with less years in the profession, whereas no clear associations with gender can be drawn^3^. It has been speculated that men and women experience burnout in different ways^22^. High workload has been shown to be associated with increased burnout levels, whereas working in the public sector and supervision and/or personal therapy has a positive effect on mental health of psychotherapists as systematically reviewed recently^3^.

While the current literature may not sufficiently explore a broad spectrum of sociodemographic characteristics, work-related factors, and behaviors such as physical activity and smartphone use that could influence psychotherapists' mental health, the present study aims to fill this gap. By investigating this broader range of variables in 2023, we aim to unearth the diverse sources contributing to mental well-being and psychological distress in psychotherapists.

## Results

### Study sample characteristics

Only psychotherapists providing information on all outcome measures were included in the final analyses (n = 502). All of these psychotherapists provided information on sociodemographic characteristics and health behaviors. With the exception of one psychotherapist providing no information on years in experience and two psychotherapists giving no information on their theoretical orientation, all work-related data were provided by the n = 502 psychotherapists.

The characteristics of the study sample are summarized in Table 1. Most participating psychotherapists were female (79.7%), between 46 and 64 years old (71.8%), lived in eastern Austria (57.2%), and had a net household income exceeding € 3000 per month (57.0%). Almost half of them were more than ten years in the profession (46.8%) and had a humanistic orientation (46.3%). Nearly all (98%) worked in independent practice. 16.7% were employed in an outpatient facility, and 7.8% in an inpatient facility. While all the participating psychotherapists provided individual psychotherapy, nearly half provided couples therapy (46.8%) and about one-fifth group therapy (20.7%). More than half of the psychotherapists (54.6%) derived all their income solely from psychotherapy, and more than one-third (36.9%) were engaged in an additional job next to psychotherapy. Physical activity recommendations of the WHO were reached by 63.7% of the participants, and the majority (50.4%) spent between one and two hours on their smartphones daily.

**Table 1:** Study sample characteristics (n = 502).

Gender	
Female, % (N)	79.7 (400)
Male, % (N)	20.3 (102)
Age in years	
≤ 45, % (N)	18.1 (91)
46–54, % (N)	35.3 (177)
55–64, % (N)	36.5 (183)
≥ 65, % (N)	10.2 (51)
Region	
Western Austria, % (N)	30.3 (152)
Eastern Austria, % (N)	57.2 (287)
Southern Austria, % (N)	12.5 (63)
Income	
< € 1000, % (N)	3 (15)
€ 1000–€ 2000, % (N)	14.7 (74)
€ 2001–€ 3000, % (N)	25.3 (127)
€ 3001–€ 4000, % (N)	25.1 (126)
> € 4000, % (N)	31.9 (160)
Years in the profession	
≤ 5, % (N)	31.1 (156)
6–10, % (N)	21.9 (110)
≥ 11, % (N)	46.8 (235)
Cluster	
Psychodynamic, % (N)	21.0 (105)
Humanistic, % (N)	46.3 (231)
Systemic, % (N)	25.1 (125)
Behavioral, % (N)	7.6 (38)
Number of patients per week	
≤ 10, % (N)	16.5 (83)
11–20, % (N)	38.8 (195)
21–30, % (N)	29.1 (146)
> 30, % (N)	15.5 (78)
Patient group	
Solely adults, % (N)	41.4 (208)
Also children and adolescents, % (N)	58.6 (294)
Practice	
Independent practice, % (N)	98.0 (492)
Outpatient facility, % (N)	16.7 (84)
Inpatient facility, % (N)	7.8 (39)
Setting	
Individual, % (N)	100 (502)
Couples, % (N)	46.8 (235)
Families, % (N)	27.5 (138)
Group, % (N)	20.7 (104
Income solely from psychotherapy, % (N)	54.6 (274)
Other job next to psychotherapy, %(N)	36.9 (185)
Physical activity	
< 3 days per week, % (N)	36.3 (182)
≥ 3 days per week, % (N)	63.7 (320)
Smartphone usage	
< 1 h per day, % (N)	33.9 (170)
1–2 h per day, % (N)	50.4 (253)
≥ 3 h per day, % (N)	15.7 (79)

Total numbers do not always sum up to n = 502 psychotherapists as not all work-related data were available for all psychotherapists. The NUTS 1 classification (Nomenclature of territorial units for statistics) was used for the classification of major socio-economic regions of Austria (Eastern Austria: Burgenland, Lower Austria, Vienna; Southern Austria: Carinthia, Syria; Western Austria: Upper Austria, Salzburg, Tyrol, Vorarlberg). Physical activity was classified following the WHO recommendations to exercise at least three days per week.

The proportion of participating male psychotherapists (20.3%) was lower compared to the total population of licensed Austrian psychotherapists (25.6%; *P* = 0.008). Compared to the total population of Austrian psychotherapists, participating psychotherapists had fewer professional experience (12.36 ± 9.438 years vs. 16.33 ± 10.921; *P* < 0.001). Also, the participating psychotherapists are not representative for the total population regarding theoretical orientation (*P* < 0.001). While humanistic psychotherapists were overrepresented in the survey (46.3% vs. 38.5% in the list), behavioral psychotherapists were underrepresented (7.6% vs. 12.3% in the list). The proportion of participating psychodynamic (21.0%) and systemic (25.1%) psychotherapists closely resembled the respective total populations of Austrian licensed psychotherapists (25.4% psychodynamic, 23.8% systemic).

### Prevalence of mental health indicators

A total of n = 42 psychotherapists (8.4%) exceeded the cut-off for clinically relevant depressive symptoms (i.e., PHQ-2 scores ≥ 3). A similar proportion (8.2%, n = 41) met the criteria of clinically relevant anxiety symptoms (GAD-2 scores ≥ 3). Symptoms of insomnia were observed in 25 (n = 5.0%) psychotherapists, as indicated by an ISI-2 score ≥ 6. High subjective stress levels (PSS-4 scores ≥ 6) were assessed in 20.5% (n = 103) of the participants. A total of n = 126 (25.1%) had a WHO-5 score ≤ 50 and where thus classified as poor-well-being group.

Mean values were M = 1.10 (SD = 1.064) for the PHQ-2, M = 0.95 (SD = 1.028) for the GAD-2, M = 2.51 (SD = 1.752) for the ISI-2, M = 3.55 (SD = 2.676) for the PSS-4 and M = 62.10 (SD = 18.09) for the WHO-5.

### Associations of mental health indicators with sociodemographic characteristics

**Figure 1:**
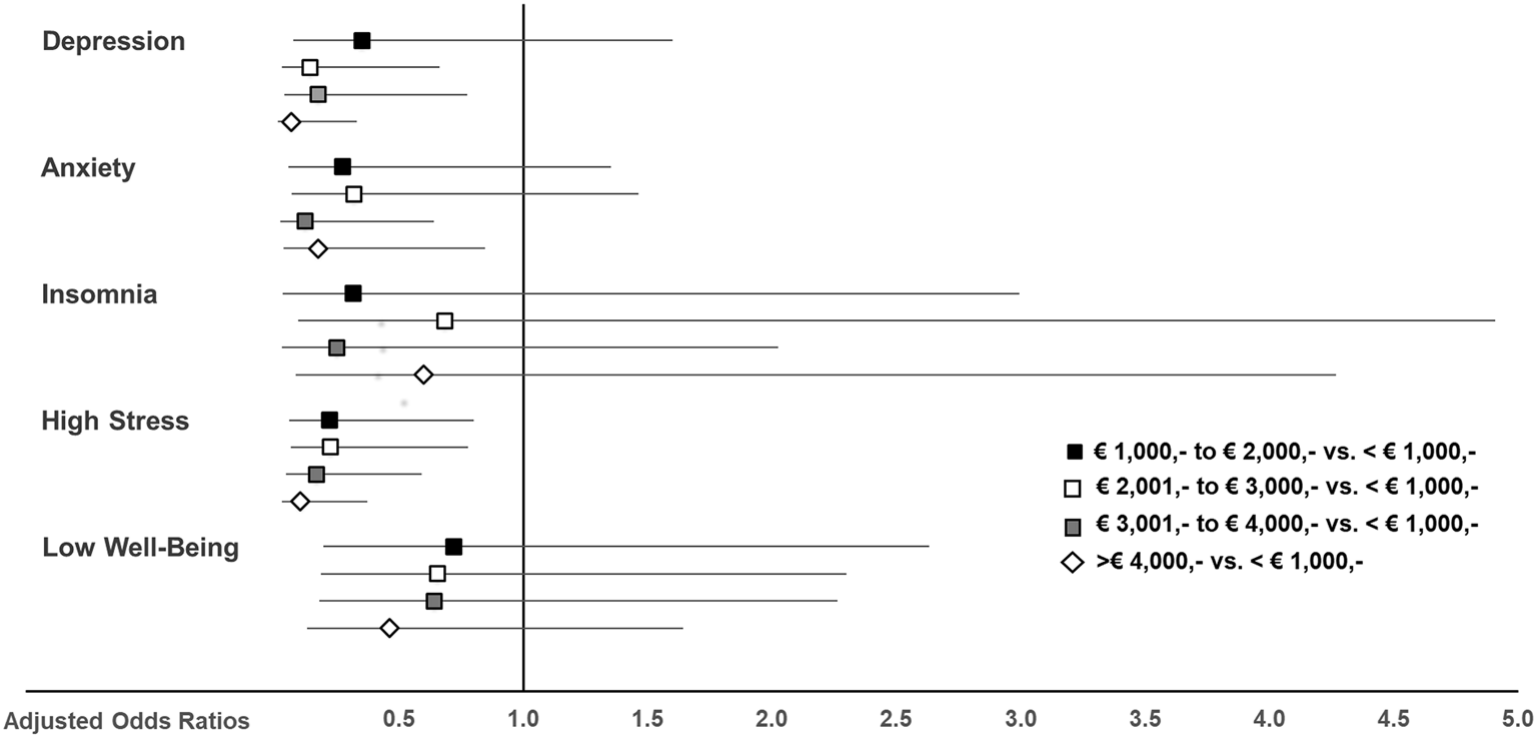
Adjusted odds ratios for clinically relevant symptoms of depression, anxiety, insomnia, high stress, and poor well-being in psychotherapists concerning net household income. An adjusted odds ratio of 1 indicates no difference. Adjusted odds ratios < 1 indicate lower relative risk in the respective income group compared to < € 1000,- net household income. 95% confidence intervals (horizontal lines) crossing 1 (vertical line) indicate no significant difference between the respective income group and < € 1000,- net household income. The multivariable logistic regression model was adjusted for gender, age, region, income, physical activity, smartphone usage, number of patients treated per week, years in the profession, patient group, therapeutic setting, facility, theoretical orientation, income dependency on psychotherapy, working in another job next to psychotherapy.

Among all sociodemographic variables investigated (gender, age, region, income), only income remained significant when all variables were considered simultaneously in the statistical model. Figure 1 demonstrates that a higher net household income decreased the odds of experiencing clinically relevant symptoms of depression, anxiety, and high stress, but not insomnia or well-being.

### Associations of mental health indicators with work-related variables

Among the work-related variables, the number of patients treated, the occupational status, the years in the profession, and the patient group were associated with mental health indicators. In contrast, no association was observed for the theoretical orientation, the therapeutic setting (group, family, couples therapy), income dependency on psychotherapy and being engaged in another job.

The number of patients was associated with the odds of experiencing high stress and poor well-being. More specifically, treating 11–20 patients per week vs. up to 10 patients per week decreased the adjusted odds for high stress to 0.485 (95% CI: 0.243, 0.969). Also, treating 21 to 30 patients per week vs. up to 10 patients per week decreased the adjusted odds for high stress to 0.248 (95% CI: 0.105, 0.583). Treating more than 30 patients per week did not change the odds of experiencing high stress, compared to treating less than ten patients per week (1.012, 95% CI: 0.449, 2.282). For poor well-being, a significant effect was only observed for treating 21 to 30 vs. up to 10 patients per week, where lower odds for poor well-being were found when a higher number of patients were treated per week (aOR: 0.337, 95% CI: 0.152, 0.748).

Practicing in an outpatient facility increased the adjusted odds for clinically relevant insomnia symptoms (aOR: 3.444, 95% CI: 1.084, 10.257) and high stress (aOR: 2.035, 95% CI: 1.087, 3.811).

More years in the profession were associated with lower adjusted odds of experiencing high-stress levels. Being a licensed psychotherapist for more than ten years decreased the odds compared to being within the first five years in the profession (0.502, 95% CI: 0.263, 0.956).

Treating not only adults but also children and adolescents decreased the adjusted odds for clinically relevant symptoms of insomnia (aOR: 0.161, 95% CI: 0.049, 0.530) and high stress (aOR: 0.446, 95% CI: 0.256, 0.775).

### Association of mental health indicators with health behaviors

**Figure 2:**
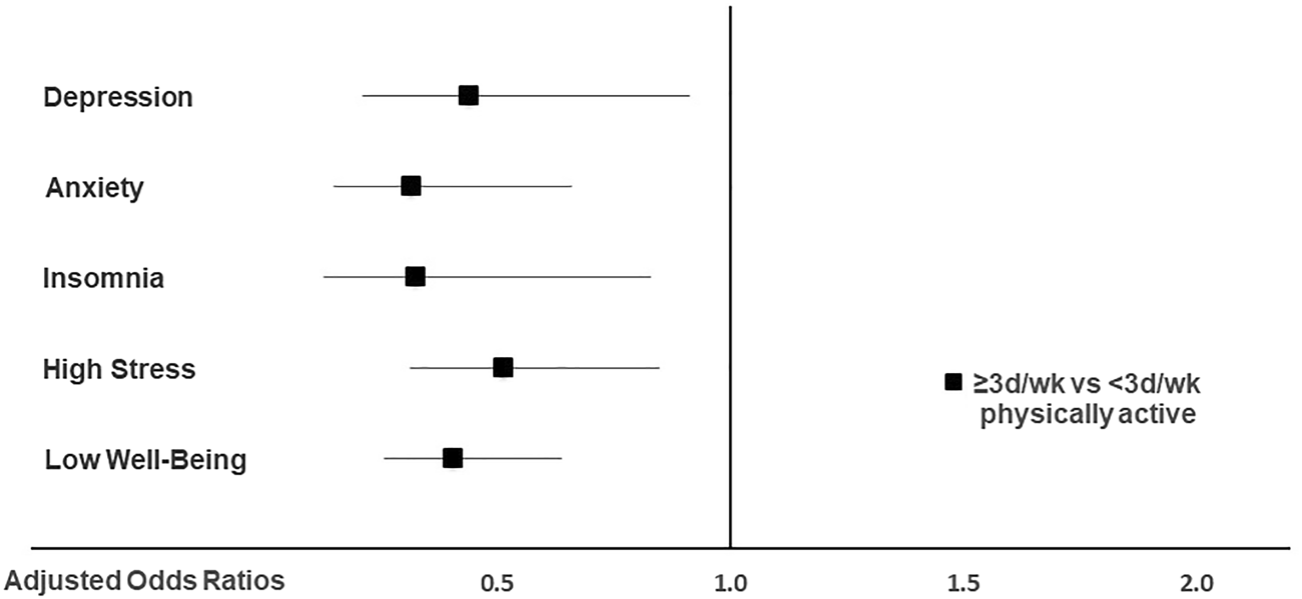
Adjusted odds ratios for clinically relevant symptoms of depression, anxiety, insomnia, high stress, and poor well-being in psychotherapists meeting WHO recommendations for physical activity vs. those not. An adjusted odds ratio of 1 indicates no difference. Adjusted odds ratios < 1 indicate lower relative risk in psychotherapists meeting WHO recommendations for physical activity vs. those not. 95% confidence intervals (horizontal lines) crossing 1 (vertical line) indicate no significant difference between psychotherapists meeting WHO recommendations for physical activity vs. those not. The multivariable logistic regression model was adjusted for gender, age, region, income, physical activity, smartphone usage, number of patients treated per week, years in the profession, patient group, therapeutic setting, facility, theoretical orientation, income dependency on psychotherapy, working in another job next to psychotherapy.

As depicted in Fig. 2, physical activity was associated with the adjusted odds of experiencing clinically relevant symptoms of depression, anxiety, insomnia, stress, and poor well-being. Psychotherapists who met the official recommendations for physical activity had lower odds of experiencing clinically relevant symptoms of all investigated mental health problems and had lower odds of poor well-being.

The risk of experiencing poor well-being was lower in psychotherapists spending 1 to 2 h per day on their smartphones compared to those spending less than 1 h per day (aOR: 0.580, 95% CI: 0.348, 0.967).

### Proportion of variance explained by the regression models

The amount of variance explained as assessed with Nagelkerke’s R^2^ was 0.231 for depressive symptoms, R^2^ = 0.215 for anxiety symptoms, R^2^ = 0.232 for insomnia symptoms, R^2^ = 0.208 for symptoms of high stress and R^2^ = 0.183 for signs of poor well-being. Results suggests a weak (well-being) to moderate (depression, anxiety, insomnia, stress) relationship between the predictors and the outcome.

## Discussion

Our study on Austrian psychotherapists identifies low income and limited physical activity as key risk factors for mental health problems, even after adjusting for various potential confounders. In addition, other work-related factors demonstrated a relationship with adverse mental health outcomes, which we will discuss in subsequent sections.

The observed prevalences for depressive, anxiety and insomnia symptoms (8.4%, 8.2%, 5.0%) were similar to those observed in our survey conducted in Austrian psychotherapists one year earlier (11.3% depression, 10.9% anxiety, 5.3% insomnia) and is considerably lower compared to the prevalences assessed in an age- and gender-matched subsample of the general population surveyed in 2022 (24.4% depression, 17.9% anxiety, 9.9% insomnia^7^).

There is limited research on the mental health of psychotherapists, leaving a significant gap in our understanding of the prevalence of mental health disorders among psychotherapists. The most extensive study conducted on mental health issues among mental health care professionals identified a higher lifetime prevalence (63%) of mental health symptoms compared to estimates in the general population (41%)^13^.

A recent meta-review of 40 systematic reviews on the mental health of healthcare professionals (doctors, nurses, and allied health care professionals) encompassing more than 3 million participants, revealed a higher prevalence of mental health issues compared to the general population^23^. Prevalences of anxiety ranged from 16 to 41%, depression from 14 to 37%, insomnia from 24 to 47%, which is clearly above the prevalences observed in psychotherapists of the current study.

In contrast to the findings of the aforementioned studies, our research suggests that psychotherapists exhibit better mental health compared to the general population. We hypothesize that this variance can be elucidated by specific characteristics inherent to Austrian psychotherapists as discussed in detail previously^7^. In brief, key factors contributing to their superior mental health include a secure social background, high professional motivation, and the ability to independently manage their time.

Among various sociodemographic factors, our study intriguingly identified income as the sole characteristic correlated with the mental health indicators depression, anxiety, and stress. When controlling for multiple variables in our model, gender and age demonstrated no significant impact. Instead, income emerged as a common denominator linked to the mentioned mental health indicators depression, anxiety, and stress. This connection between lower household income and heightened risk of mental disorders is consistent with previous studies^24^ and has been observed in a recent study in the Austrian general population^20^.

Our findings further solidify the assumption that income disparities account significantly for the comparatively better mental health observed in psychotherapists versus the general population^7^. For instance, a net household income below € 2000 per month constitutes a risk factor for mental health disorders in the general population, with over 40% belonging to this income category^20^. Conversely, psychotherapists within this low-income bracket make up a considerably smaller proportion, less than half at 17.7%.

Despite lower income being less prevalent among psychotherapists compared to the general population, income variability still constitutes a notable source of mental health diversity within the profession. As substantiated by previous research, financial concerns remain a primary stressor for psychotherapists in Germany^25^. The economic circumstances of psychotherapists necessitate further attention within the context of professional policy, given the demonstrated direct association with their mental health in our study. However, we must also consider that lower income could indicate underlying issues linked to mental health challenges, including poorly integrated personality traits, potentially limiting work capacity, and resulting in lower earnings. This notion aligns with a 2022 mixed methods study segregating a sample of Austrian psychotherapists into well-being categories per WHO-5 standards. Therapists within the lower well-being category reported increased interpersonal difficulties and strain across domains such as 'excessive demand', 'negative emotions', and 'physical health' while serving fewer patients weekly^26^. Regardless of the directionality of this relationship—whether income (perhaps due to low caseloads, see below) influences mental health or the converse—the existence of a psychotherapist subgroup with lower earnings and mental health challenges warrants attention due to potential implications on the overall quality of therapeutic services provided. Further monitoring and intervention strategies are therefore crucial for this subset of psychotherapists.

Among the work-related variables, years in the profession, patient numbers, patient group, and occupation were associated with mental health indicators.

Our study reveals a correlation between the number of patients treated by psychotherapists and their mental health status, here stress and well-being. Specifically, therapists managing a medium-sized patient load—ranging from more than 10 to fewer than 30 weekly—exhibited the best mental health indicators. This range could signify an ideal 'patient sweet spot' for maintaining optimal stress levels and well-being. Conversely, a caseload exceeding 30 patients per week seems to negate these benefits, likely due to the inherent strain of managing such a volume, a scenario corroborated by prior research linking heavy workloads to increased burnout rates^27–32^. Assuming a medium patient load is optimal, one possible explanation is that managing a certain number of patients propels therapists into a 'flow' state. This condition is associated with increased competency and reduced stress, potentially fueled by a sense of effectiveness, engagement, and fulfillment from helping numerous patients. Alternatively, less-stressed therapists might inherently manage larger caseloads more effectively, thanks to superior stress management skills, personal resilience, or efficient professional practices.

Interestingly, therapists handling fewer than 11 patients exhibited worse stress and well-being compared to their peers with 21–30 patients per week. This discrepancy might stem from the pressures of maintaining a private practice with insufficient patient numbers, leading to increased stress levels. Alternatively, self-aware therapists might intentionally limit their patient load to manage stress. There might also be other confounding variables for low patient numbers and psychological distress symptoms, such as the presence of other potentially stressful obligations, such as responsibility for children, relatives in need of care, own health restrictions, etc. These external factors might contribute to the observed stress levels, independently or in combination with patient load. However, these interpretations remain conjectural, necessitating further investigations to substantiate either hypothesis.

Similar to the lower odds of experiencing high-stress levels in psychotherapists with higher professional experience (> 10 years in the profession), previous research indicates that less experienced psychotherapists are more prone to develop emotional exhaustion and burnout syndromes^28,33–35^. However, results should be interpreted with caution, as only psychotherapists (still) active were invited to participate. Thus, there might be some selection bias toward psychotherapists who cope well with their professional demands in the more experienced group, as those being emotionally exhausted might have quit their psychotherapeutic work within the first years in the profession.

Working in an outpatient facility enhanced the odds of experiencing insomnia and high stress. This finding might be explained by lower opportunities to control the work environment compared to private psychotherapists` practices. Working in facilities might also go along with higher levels of bureaucracy and a need to follow strict protocols^36^. Self-employment is associated with higher job satisfaction^37^, likely due to higher autonomy, work schedule flexibility, job security, and skill utilization compared to organizational employment^38^. In this regard, previous studies also observed associations between work settings and burnout levels^30,32,35,39,40^.

Our result suggests that treating not only adults but also children and adolescents in the same clinical setting is associated with decreased odds for clinically relevant symptoms of insomnia and high stress. This might seem counter-intuitive initially, as one might expect that dealing with younger patients, who can present different or additional challenges, might increase stress. A possible explanation might hinge on the perception of older patients as being more seriously ill yet less amenable to treatment than younger patients^41^. Notably, a meta-analysis reinforces this idea, suggesting the heightened effectiveness of psychotherapies in young adults^42^. Engaging with younger patients allows clinicians to intervene and curtail psychological issues early, fostering a sense of achievement that lowers stress levels. Additionally, the varied interaction with a diverse patient population and the zest and spontaneity that children and adolescents often bring into therapy could safeguard therapists against the monotony and boredom that significantly contribute to burnout^43^. More detailed research needs to be conducted, including qualitative interviews with the therapists to understand their experiences and perceptions about working with different age groups.

Among various health behaviors, physical activity merits special consideration. Adherence to the World Health Organization's (WHO) recommendations for physical activity was inversely associated with all evaluated mental health problems, suggesting that regular exercise could be a protective factor for psychotherapists' mental health. Previous empirical studies corroborate this, showing lower odds for mental disorders in physically active individuals^18^, less exhaustion, higher professional efficacy^44^, higher quality of life, and lower rates of burnout^45^. Although mental health care professionals often recommend physical activity as an essential self-care practice to their patients^8,46^, our findings suggest their commitment to physical activity does not surpass that of the general population. In our study, the proportion of psychotherapists meeting the WHO recommendations (63.7%) was comparable to that observed in the general population (59.8%)^18^. Overall, the results of the current study contribute to the knowledge about self-care behaviors among psychotherapists in highlighting the importance of adequate physical activity to foster mental health. Nevertheless, due to the cross-sectional nature of the study the directionality of the association between physical activity and mental health remains unclear. Therefore, it has to be noted that lower physical activity could also be a consequence or symptom of impaired mental health and not the cause.

The current findings suggest a varying but generally moderate strength of the relationship between the predictors and the respective outcomes, ranging from weak in the case of well-being (Nagelkerke’s R^2^ = 0.183) to moderate in the domains of depression (Nagelkerke’s R^2^ = 0.231), anxiety (Nagelkerke’s R^2^ = 0.215), insomnia (Nagelkerke’s R^2^ = 0.232), and stress (Nagelkerke’s R^2^ = 0.208). The identified relationships underscore that while the studied predictors contribute significantly to the understanding of depressive, anxiety, insomnia, and stress symptoms, there are likely other factors not accounted for in the model. Further research and consideration of additional factors are warranted for a comprehensive understanding.

This study has several limitations. First, all data were derived solely from self-reports and not objectively measured. Objectively assessed health behavior data, for example by measuring screen time and physical activity on the smartphone, and objectively assessed mental health problems via structured or standardized interviews would have provided results of higher validity. A further limitation is the moderate internal consistency of the PHQ-2 (Cronbach’s α = 0.58), GAD-2 (Cronbach’s α = 0.58) and ISI-2 (Cronbach’s α = 0.62) in the current psychotherapists’ sample. The same scales applied in the general population one year earlier yielded higher consistency scores (PHQ-2: α = 0.81, GAD-2: α = 0.84, ISI-2: α = 0.78)^7^. The lower consistency scores observed in the psychotherapists' sample compared to the general population sample could be attributed to differences in the psychological characteristics, experiences, or interpretation tendencies of psychotherapists compared to the general population. It's possible that the constructs being measured by these scales operate differently or are perceived differently within the context of a sample of psychotherapists, leading to lower correlations among the scale items. Further investigation would be needed to pinpoint the specific factors contributing to this discrepancy. Further, the online conduction of the study might have caused some respondent bias, i.e., towards those being more familiar with digital technologies. This is supported by participation of psychotherapists with less years in the profession and therefore probably younger ones than the general population of Austrian psychotherapists. Due to the unique education system for psychotherapists in Austria (i.e., a high number of hours required for self-therapy, obligation to engage in supervision when working as a psychotherapist), results might not generalize to other countries with other education or healthcare systems.

## Conclusions

This study suggests that heterogeneity in mental health in psychotherapists may to a certain degree be attributable to health behaviors (i.e., physical activity), sociodemographic (i.e., income), and work-related characteristics (i.e., years in the profession, patient numbers, and work settings). Specific attention should be directed towards psychotherapists in the early years of their careers, those earning lower incomes, individuals lacking regular physical activity, and those employed in healthcare institutions. Enhancing their self-care behaviors could diminish the risk of developing mental health disorders, thereby maintaining their overall well-being.

Given the imperative for psychotherapists to deliver high-quality care, improving their mental health is vital for the therapists themselves and consequential for their patients. We recommend implementing regular outcome monitoring for quality assurance and a systematic selection process based on measures such as comprehensive mental health screening, personality testing, and longitudinal assessments throughout the training process that considers the psychological suitability of potential psychotherapy students.

Despite our study elucidating certain relationships between health behaviors, sociodemographic factors, and work-related characteristics, further research must show whether these associations show a cause-and-effect relationship.

## Methods

### Design

Licensed Austrian psychotherapists were surveyed online between April 3, 2023, and June 5, 2023. The survey was set up in Research Electronic Data capture (REDcap)^47^. The survey link was sent to psychotherapists registered in the official list of licensed psychotherapists provided by the Austrian Federal Ministry of Social Affairs, Health, Care and Consumer Protection. Of the > 11,000 psychotherapists registered in April 2023, about 7000 provided a valid e-mail address and thus could be contacted by e-mail. Moreover, the Austrian Federal Association for Psychotherapy (ÖBVP) informed their members (≈ 5000 psychotherapists) about the study and provided the link to the survey via their newsletters. Psychotherapists received no incentives, and participation was voluntary and anonymous. All participants gave electronic informed consent to participate and complete the questionnaire. The present study was carried out in accordance with the Declaration of Helsinki and approved by the data protection office and the Ethics Committee of the University for Continuing Education Krems, Austria (Ethical no. EK GZ 11/2021-2024).

## Measures

### Sociodemographic variables

Psychotherapists were asked about their gender (female, male, diverse), age (in years), region, and net household income per month (< € 1000, € 1000–€ 2000, € 2001–€ 3000, € 3001–€ 4000, > € 4000). The categorization of the income levels was in accordance with previous studies in the Austrian general population^17,19,20^ to enable comparisons.

### Work-related variables

Participants were further asked about their years in the profession, their theoretical orientation (psychodynamic, humanistic, systemic, behavioral), the number of patients treated on average per week, the patient group (adults, children, and adolescents), the treatment format (individual, couples, families, groups), and the practice type (private, outpatient facility, inpatient facility). Finally, they were asked whether their income relies solely on psychotherapeutic treatments and whether they are engaged in another job next to psychotherapy.

### Health behaviors

As health behaviors, physical activity (days of at least 60 min of physical activity per week) and smartphone use (< 1h/d, 1-2h/d, 3-4h/d, 5-6h/d, 7-8h/d, > 8h/d) were assessed by self-report. Following recent recommendations of the World Health Organization (WHO) to exercise at least three days per week^48^, physical activity was divided into two groups (< 3 days of physical activity per week vs. ≥ 3 days of physical activity per week). Smartphone usage was grouped among psychotherapists using the smartphone for less than 1h/d, those spending 1 to 2h/d on their smartphones, and those spending at least 3 h daily on their smartphone. Previous data suggest that spending more than 2 h/day on the smartphone enhances the risk of poor health outcomes^49^.

### Depressive symptoms (PHQ-2)

Symptoms of depression during the past two weeks were assessed with the two-item version of the depression module of the Patient Health Questionnaire^50^. The PHQ-2 sets depressive symptoms on a four-point scale from 0 (not at all) to 3 (nearly every day) by self-report. The total score ranges from 0 to 6, with higher scores suggesting more severe depressive symptoms. Scores ≥ 3 have been reported to be indicative of moderate (clinically relevant) depressive symptoms^51^. Internal consistency (Cronbach's alpha) was α = 0.58 in the current sample.

### Anxiety (GAD-2)

The General Anxiety Disorder scale^52^ short version was applied to measure anxiety symptoms. The GAD-2 scale assesses symptoms of generalized anxiety over the past two weeks on a four-point self-rated Likert scale from 0 (not at all) to 3 (nearly every day), yielding sum scores from 0 to 6. Total scores of at least 3 points indicate clinically relevant anxiety symptoms^53^. Cronbach's alpha was α = 0.58 in the sample at hand.

### Insomnia (ISI-2)

A short version of the Insomnia Severity Index (ISI) was applied to assess sleep quality over the last two weeks^54^. The two items of the ISI-2 measure satisfaction/dissatisfaction with sleep and interferences with daily functioning by self-reporting on a five-point scale from 0 to 4. The total scores range from 0 to 8, with a cut-off score of ≥ 6 indicating clinically relevant insomnia symptoms^55^. Cronbach's alpha was α = 0.62 in the present psychotherapists` sample.

### Perceived stress (PSS-4)

The perceived stress levels during the last two weeks were assessed with the short version of the Perceived Stress Scale. The four items of the PSS-4 assess the experienced stress on a five-point Likert scale from 0 (never) to 4 (very often) by self-report^56^. Items 2 and 3 need to be reversed coded before the total scores can be calculated by summing up the scores of all items. Total scores range from 0 to 16, with higher scores indicating higher stress levels. A cut-off of at least 6 points has been suggested to represent high subjective stress levels^57^. Cronbach's alpha was α = 0.72 in the present sample.

### Well-being (WHO-5)

The subjective well-being experienced during the past two weeks was assessed with the World Health Organization Well-being Index (WHO-5)^58^. The five positively phrased questions on the well-being of the WHO-5 are self-rated on a six-point Likert scale from 0 (none of the time) to 5 (all of the time). The raw total scores ranged from 0 to 25 and were translated into a percentage scale from 0 (absence of well-being) to 100 (maximal well-being)^59^. Exceeding a cut-off of 50 points has been suggested to indicate good well-being^60^. Cronbach's alpha was α = 0.87 in the current psychotherapist's sample.

### Statistical analyses

Descriptive statistics were conducted to describe sociodemographic, work-related, and health behavior characteristics.

Differences in sociodemographic and professional traits between participating psychotherapists and the overall population of licensed Austrian psychotherapists were evaluated using chi-squared tests and t-tests for independent samples.

To analyze the associations of mental health indicators with the assessed sociodemographic, work-related, and health behavioral characteristics, multivariable binary logistic regressions were applied.

The health indicators investigated comprised clinically relevant symptoms of depression, anxiety, insomnia, stress, and poor well-being. The sociodemographic variables were age, gender, region, and income. Work-related variables comprised years in profession, patient numbers, theoretical orientation, patient group, setting, income dependency on psychotherapy, engaging in another job next to the psychotherapeutic work, and being employed in an outpatient/inpatient facility. As health behaviors physical activity and smartphone usage were incorporated in the statistical analyses.

All the above-described sociodemographic, work-related, and health behavior variables were included as predictors to explore the independent contribution of the investigated variables. The mental health variables functioned as dependent variables in the multivariable binary logistic regression model.

Adjusted odds ratios (aOR) and 95% confidence intervals (CIs) were estimated to assess the statistical uncertainty. The amount of explained variance was calculated by Nagelkerke’s *R*^2^^61^. Statistical analyses were performed using SPSS version 26 (IBM Corp, Armonk, NY, USA). *P*-values of < 0.05 were considered statistically significant (2-sided tests).

### Ethical approval

The study involving human participants was reviewed and approved by the Ethics Committee of the University for Continuing Education Krems, Austria (Ethical no.: EK GZ 11/2021–2024). The participants provided their written informed consent to participate in this study.

## Data Availability

The datasets for this study will be made available from the corresponding author upon reasonable request.
